# Field Monitoring and Analysis of the Vibration of Stay Cables under Typhoon Conditions

**DOI:** 10.3390/s20164520

**Published:** 2020-08-12

**Authors:** Jian Guo, Xujiang Zhu

**Affiliations:** Institute of bridge Engineering, Zhejiang University of Technology, Hangzhou 310023, China; river@zjut.edu.cn

**Keywords:** sea-crossing bridge, field measurement, stay cable, wavelet-matrix transform, wavelet packet, wind characteristics, nonstationary

## Abstract

Structural health monitoring systems provide many advantages for full-scale measurements in bridge monitoring. In this study, a strong landing typhoon event recorded at the Jintang Bridge (Zhejiang Province, China) in 2019 was selected to study the nonstationary wind and cable vibration characteristics. To study the characteristics of the recorded typhoon, the time-varying mean wind was extracted based on the adaptive method of the wavelet-matrix transform. The nonstationary characteristics of Typhoon Lekima, including the turbulence intensity, gust factor, and fluctuating wind power spectral density, were analyzed and compared with the stationary model characteristics of a typhoon, and the typical characteristics and parameters were obtained. In addition, the measured vibration response of the cables was analyzed. The vibration characteristics of the cables and the energy distribution of the wind speed wavelet packet were investigated. The vibrations at different positions were compared. A power spectrum analysis and a wavelet packet energy analysis of the cable were performed. The results of this study can be used as a basis for wind-resistant design and performance evaluation of bridges under similar operational conditions.

## 1. Introduction

With the demand for economic development, China has built many long-span bridges, seven of which are among the ten longest main-span, cable-stayed bridges in the world [1]. However, with the increase in bridge span, the structure has become flexible, the basic natural vibration period has reached >10 s, and the damping has decreased, making such bridges more sensitive to wind-induced vibration [2]. Especially for a cable-stayed bridge, the stay cables are vulnerable components, and the buffeting effects of the stay cables are more serious. Consequently, the wind load has become the most important control factor affecting structural safety [3]. Therefore, field monitoring of the wind environment [4,5,6] and structure dynamic response [7] has become an important topic. Recording wind environment measurements and performing a buffeting statistical analysis of the measured data constitute the most effective way to understand the wind characteristics of a region.

Previous studies of the wind characteristics of typhoons have focused on a stationary wind model hypothesis. Early investigations were conducted by Davenport [8], Kaimal et al. [9], Panofsky and Singer [10], Harris [11], and Shiotani and Iwatani [12]. These researchers have done considerable work in the field of wind characteristics measurement and have developed their own wind field models, and their results have been widely used in wind engineering specifications in various countries [13,14,15]. Because typhoons have obvious nonstationary characteristics that differ from those of stationary wind models, researchers have turned their attention to the study of nonstationary random wind fields under extreme weather conditions [16,17,18,19]. Based on the above premise, various nonstationary characteristics methods have been devised and their feasibility has been demonstrated. Proposed methodologies based on empirical mode decomposition and the discrete wavelet transform have also been closely followed and further investigated in many more recent studies, including those by Xu and Chen [20], Tao et al. [21], and Huang and Chen [22], which mostly address the nonstationary characteristics of typhoons in field measurements. However, for natural wind with strong randomness, it is very difficult to formulate universal standards and use them in the wind-resistant design of various engineering structures. The most common problem is that the design is too conservative to ensure the safety of the structures or that the lack of research on wind characteristics leads to insufficient wind-resistant design and structural safety problems. Therefore, it is necessary to conduct measured research in targeted cases.

In this paper, we propose that the wavelet-matrix transform (WMT) be directly applied to the nonstationary characteristics of typhoons [23,24]. Because any complex function can be described by a dense equidistant sampling data series, the discrete WMT is still general. Compared with the nonstationary characteristics analysis method mentioned above, WMT entails analyzing the essence of the wavelet transform from another aspect. It can be used to directly understand the decomposition and reconstruction of the orthogonal wavelets from the time domain, and the calculation of wavelet orthogonal decomposition through a matrix analysis is faster and easier to understand. In this study, Typhoon Lekima, which landed on the Jintang Bridge in 2019, was taken as the background object. Based on the measured data of Typhoon Lekima collected by the structural health monitoring system (SHMS), a nonstationary wind speed model suitable for the nonstationary wind speed data and the corresponding time-varying mean wind speed and fluctuating wind speed extraction method were established. Comparison with the wind resistance specifications of Chinese bridges provides a basis for the wind resistance safety assessment of the bridge based on the SHMS and provides a reference for other large structures in similar marine environments, especially for other sea-crossing bridges in Zhejiang Province.

## 2. Case Study

**Figure 1:**
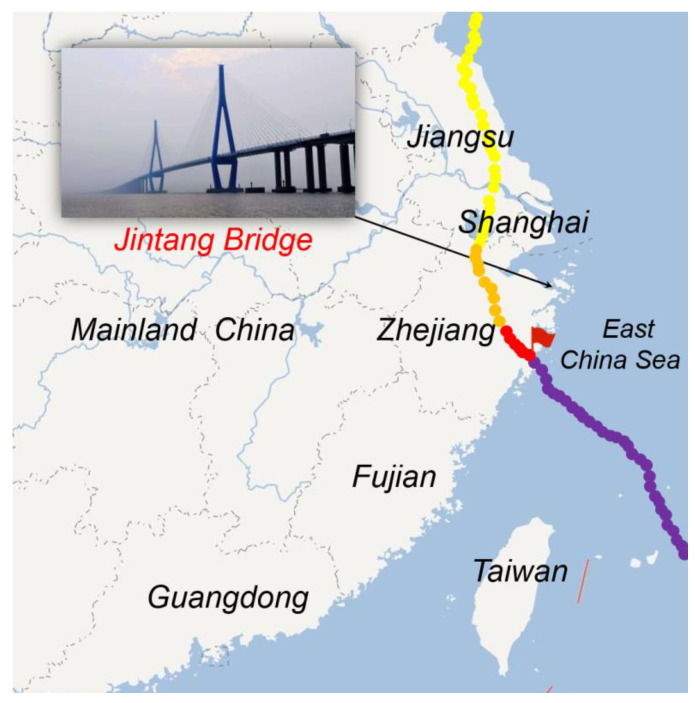
Moving track of Typhoon Lekima.

The Jintang Bridge in China is a long cable-stayed bridge carrying a dual four-lane highway on the upper level of the bridge deck. The overall length and the main span of the bridge are 21.029 km and 620 m, respectively. The height of its two towers is 204 m, as measured from the base level to the tower saddle. The width of the bridge deck is 30.1 m. The bridge was opened to traffic in 2009. It is also located in a typhoon-prone area in the northwest of the Pacific Ocean. Typhoon Lekima formed as a tropical depression at the east of Luzon, Philippines, on 4 August 2019. It directly landed at Wenling City, Zhejiang Province, at 01:45 a.m. (UTC+8) on 10 August 2019, with a maximum wind force near the center of 52 m/s, and it arrived at Shanghai at 00:00 a.m. on 11 August 2019. Finally, it crossed Jiangsu Province on 10 August 2019 and moved to Shandong and its adjacent waters, from where Lekima transformed into an extratropical cyclone. From 03:00 p.m. to 05:00 p.m. on 10 August 2019, it passed the site of the Jintang Bridge. Figure 1 shows the route of Typhoon Lekima and the location of the Jintang Bridge.

**Figure 2:**
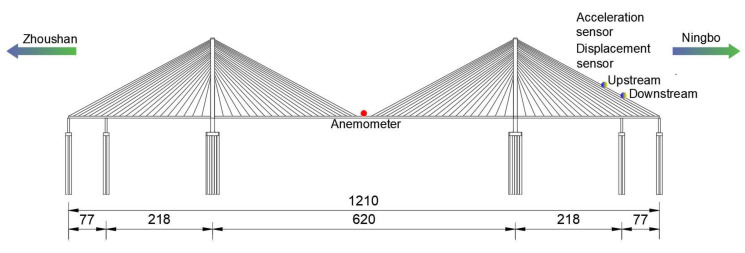
Sensor layout on the Jintang Bridge (in units of meters).

**Figure 3:**
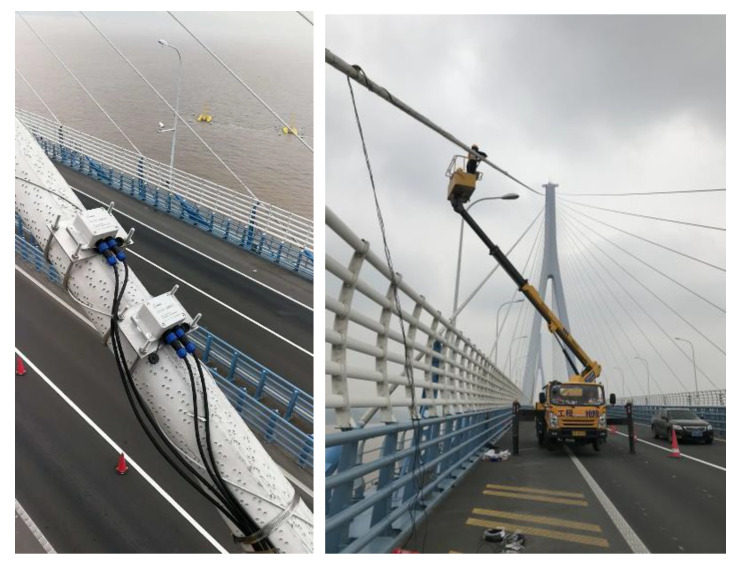
Sensor field installation on the Jintang Bridge.

**Figure 4:**
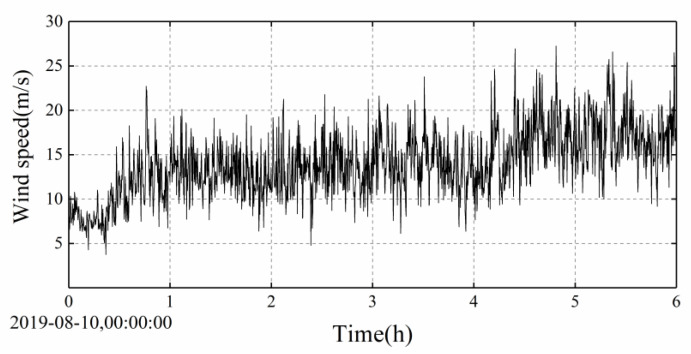

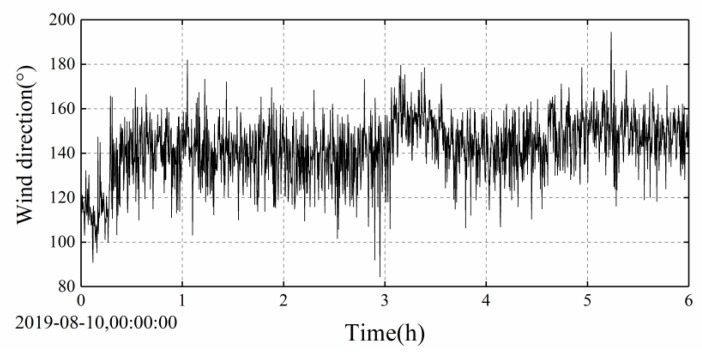
Measured wind samples during Typhoon Lekima.

At present, the Jintang Bridge is equipped with an overall SHMS, which is used to evaluate the health status of the bridge by monitoring the wind environment, temperature distribution, traffic distribution, bridge vibration response, and other bridge working environments. However, the existing cable vibration monitoring sensor of the Jintang Bridge is a unidirectional acceleration sensor, which can only monitor the vibration in the plane of the cables, while the actual vibration is an in-plane and out-of-plane common vibration. Therefore, to fully understand the wind-induced vibration response of the stay cables, a two-way acceleration sensor and a displacement sensor were added to the existing SHMS (see Figure 2). Four sensors were installed on the longest stay cable near the Ningbo side. One sensor can monitor the in-plane and out-of-plane vibration of the stay cable. Figure 3 shows the field installation of the sensor, and Table 1 lists the technical standard of the wireless acceleration node TZT3805. The sampling frequency of the anemometer was 32 Hz. In this study, the wind speed data collected by the anemometer on the Jintang Bridge and the data collected by the sensor on the stay cable were taken as the research objects. The wind speed samples during the landing period of the typhoon were selected for analyzing the wind characteristics and the vibration characteristics of the stay cable. Data from Typhoon Lekima were selected from 00:00 a.m. to 06:00 a.m. on 10 August. As can be seen from Figure 4, the wind speed time history of the strong wind samples has clear time-varying and local mutation characteristics.

## 3. Wavelet-Matrix Transform

### 3.1. Daubechies Wavelet and Mallat Matrix

Wavelet analysis can be used to decompose nonstationary processes in the time–frequency domain. Except for the explicit formula of the Harr wavelet, other wavelet functions have no explicit expression. Let f(t) be a signal in the time domain (−∞,∞). We call ψ(t) the mother wavelet. Wavelets are generated from the mother wavelet by translation and dilation as follows:

(1) $$\psi_{a,b}(t)=\frac{1}{\sqrt{a}}\psi (\frac{t-b}{a})$$

where *a* is a dilation parameter and *b* is a translation parameter. The wavelet transform of the signal f(t) is defined as

(2) $$W_{f}(a,b)=\int_{-\infty }^{\infty }f(t)\cdot \psi_{a,b}(t)dt$$

The definition of a wavelet is far from this simple, as only a wavelet family with orthogonal characteristics can decompose the function f(t); that is, it can be decomposed into a series of orthogonal wavelet combinations, and the result is unique. Only in this way can the wavelet be reconstructed back to the original function.

We consider the Daubechies (DB4) wavelet matrix as an example. It has four supporting points. We show the scale matrix and wavelet matrix of DB4. Through multiscale decomposition, we can perform a multiresolution analysis of the wavelet matrix. Wavelet matrix analysis entails decomposing each layer one by one according to the scale and wavelet coefficients and displaying the amplitude of the wavelet and scale of each layer step by step. In this process, the number of signal points in each layer should be halved. According to the above method, the matrix  A[8,8] can be constructed as follows:

$A[8,8]=\left[\begin{array}{cccccccc} h_{0} & h_{1} & h_{2} & h_{3} & 0 & 0 & 0 & 0\\ 0 & 0 & h_{0} & h_{1} & h_{2} & h_{3} & 0 & 0\\ 0 & 0 & 0 & 0 & h_{0} & h_{1} & h_{2} & h_{3}\\ 0 & 0 & 0 & 0 & 0 & 0 & h_{0} & h_{1}\\ g_{0} & g_{1} & g_{2} & g_{3} & 0 & 0 & 0 & 0\\ 0 & 0 & g_{0} & g_{1} & g_{2} & g_{3} & 0 & 0\\ 0 & 0 & 0 & 0 & g_{0} & g_{1} & g_{2} & g_{3}\\ 0 & 0 & 0 & 0 & 0 & 0 & g_{0} & g_{1} \end{array}\right]\begin{array}{c} \mathrm{First}\;\mathrm{phase}\;\mathrm{scale}\;\mathrm{waveform}\\ \mathrm{Second}\;\mathrm{phase}\;\mathrm{scale}\;\mathrm{waveform}\\ \mathrm{Third}\;\mathrm{phase}\;\mathrm{scale}\;\mathrm{waveform}\\ \mathrm{Fourth}\;\mathrm{phase}\;\mathrm{scale}\;\mathrm{waveform}\\ \mathrm{First}\;\mathrm{phase}\;\mathrm{wavelet}\;\mathrm{waveform}\\ \mathrm{Second}\;\mathrm{phase}\;\mathrm{wavelet}\;\mathrm{waveform}\\ \mathrm{Third}\;\mathrm{phase}\;\mathrm{wavelet}\;\mathrm{waveform}\\ \mathrm{Fourth}\;\mathrm{phase}\;\mathrm{wavelet}\;\mathrm{waveform} \end{array}$

Here, the first four row’s elements are scale coefficients, and the last four row’s elements are wavelet coefficients. For example, the first row’s elements consists of four scale coefficients $(h_{0},h_{1},h_{2},$ and $h_{3})$ and four zeros on the right, which is the scale coefficient in the first phase; the second row’s elements is a shift of the first row of data $(h_{0},h_{1},h_{2},$ and $h_{3})$ to the right by two columns, and the rest are all zeros, which is the scale coefficient in the second phase; and so on. When Row 4 shifts Row 3’s data to the right by two columns, data truncation occurs, and there are two columns of data that are out of the range of the eighth-order square matrix. As a result, there are only two nonzero data values in Row 4. The law of wavelet coefficients is similar to that of scale coefficients. In the fifth row’s elements, there are four wavelet coefficients $(g_{0},g_{1},g_{2},$ and $g_{3})$ and four zeros on the right, which is the wavelet coefficient in the first phase. The wavelet coefficients in the second, third, and fourth phases are similar, shifting two columns to the right.

The DB4 wavelet scale coefficient and the wavelet coefficient are given by Zhang [25]. The four coefficients of scale are

(3) $$h_{0}=\frac{1+\sqrt{3}}{4\sqrt{2}};h_{1}=\frac{3+\sqrt{3}}{4\sqrt{2}};h_{2}=\frac{3-\sqrt{3}}{4\sqrt{2}};h_{3}=\frac{1-\sqrt{3}}{4\sqrt{2}}$$

and $h_{0}^{2}+h_{1}^{2}+h_{2}^{2}+h_{3}^{2}=1$. This meets the condition of unitary.

The corresponding four wavelet coefficients are

(4) $$g_{0}=h_{3};g_{1}=-h_{2};g_{2}=h_{1};g_{3}=-h_{0}$$

and $g_{0}+g_{1}+g_{2}+g_{3}=0$, which satisfies the condition that the wavelet integral is zero.

The original signal can be decomposed into Y[8] by multiplying the A[8,8]·F[8] matrix, including the scale amplitude $C[4]={\{c}_{0}{,c}_{1}{,c}_{2}{,c}_{3}\}$ and the wavelet amplitude $D[4]={\{d}_{0}{,d}_{1}{,d}_{2}{,d}_{3}\}$.

(5) $$\left[\begin{array}{cccccccc} h_{0} & h_{1} & h_{2} & h_{3} & 0 & 0 & 0 & 0\\ 0 & 0 & h_{0} & h_{1} & h_{2} & h_{3} & 0 & 0\\ 0 & 0 & 0 & 0 & h_{0} & h_{1} & h_{2} & h_{3}\\ 0 & 0 & 0 & 0 & 0 & 0 & h_{0} & h_{1}\\ g_{0} & g_{1} & g_{2} & g_{3} & 0 & 0 & 0 & 0\\ 0 & 0 & g_{0} & g_{1} & g_{2} & g_{3} & 0 & 0\\ 0 & 0 & 0 & 0 & g_{0} & g_{1} & g_{2} & g_{3}\\ 0 & 0 & 0 & 0 & 0 & 0 & g_{0} & g_{1} \end{array}\right]\cdot \left(\begin{array}{c} f_{0}\\ f_{1}\\ f_{2}\\ f_{3}\\ f_{4}\\ f_{5}\\ f_{6}\\ f_{7} \end{array}\right)=\left(\begin{array}{c} y_{0}\\ y_{1}\\ y_{2}\\ y_{3}\\ y_{4}\\ y_{5}\\ y_{6}\\ y_{7} \end{array}\right)=\left(\begin{array}{c} c_{0}\\ c_{1}\\ c_{2}\\ c_{3}\\ d_{0}\\ d_{1}\\ d_{2}\\ d_{3} \end{array}\right)$$

If we want to reconstruct the original function according to the scale and wavelet coefficients, we can use Equation (5), but the key is whether the matrix *A* is orthogonal. The verification is as follows:

(6) A·F=Y

(7) $$A^{T}\cdot Y=F_{1}$$

As shown in the matrix, *A* is not an orthogonal matrix and needs to be modified as follows:

(8) $$A^{T}\cdot A=\left[\begin{array}{cccccccc} 0.25 & 0.433 & & & & & & \\ 0.433 & 0.75 & & & & & & \\ & & 1 & & & & & \\ & & & 1 & & & & \\ & & & & 1 & & & \\ & & & & & 1 & & \\ & & & & & & 1 & \\ & & & & & & & 1 \end{array}\right]$$

If we move the two columns of data truncated during the shift of Rows 4 and 8 to the first and second columns of the corresponding rows circularly, then, equivalent to the original data, the eighth and ninth data entries are not 0, but $f_{8}=f_{0};f_{9}=f_{1}$ and $g_{2}$ and $g_{3}$ move to Columns 1 and 2 in Row 4, which is similar to moving $g_{2}$ and $g_{3}$ to Columns 1 and 2 of Row 8. Such circularly shifted matrices are called B matrices. B matrices are orthogonal matrices:

(9) $$B=\left[\begin{array}{cccccccc} h_{0} & h_{1} & h_{2} & h_{3} & 0 & 0 & 0 & 0\\ 0 & 0 & h_{0} & h_{1} & h_{2} & h_{3} & 0 & 0\\ 0 & 0 & 0 & 0 & h_{0} & h_{1} & h_{2} & h_{3}\\ h_{2} & h_{3} & 0 & 0 & 0 & 0 & h_{0} & h_{1}\\ g_{0} & g_{1} & g_{2} & g_{3} & 0 & 0 & 0 & 0\\ 0 & 0 & g_{0} & g_{1} & g_{2} & g_{3} & 0 & 0\\ 0 & 0 & 0 & 0 & g_{0} & g_{1} & g_{2} & g_{3}\\ g_{2} & g_{3} & 0 & 0 & 0 & 0 & g_{0} & g_{1} \end{array}\right]$$

(10) $$B^{T}\cdot B=E[8,8]$$

The improved DB4 wavelet matrix is represented by a square matrix *B*, and its inner part is divided into two matrices: the upper *H* is called the “scale matrix” and the lower *G* is called the “wavelet matrix.” They are all rectangular matrices:

(11) $$B[8,8]=\left(\begin{array}{c} H[4,8]\\ G[4,8] \end{array}\right)$$

Decomposing the original data F[8] to obtain the “scale time spectrum $C_{1}[8]$” and the “wavelet time spectrum $D_{1}[8]$” gives

(12) $$\left[\begin{array}{cccccccc} h_{0} & h_{1} & h_{2} & h_{3} & 0 & 0 & 0 & 0\\ 0 & 0 & h_{0} & h_{1} & h_{2} & h_{3} & 0 & 0\\ 0 & 0 & 0 & 0 & h_{0} & h_{1} & h_{2} & h_{3}\\ h_{2} & h_{3} & 0 & 0 & 0 & 0 & h_{0} & h_{1}\\ g_{0} & g_{1} & g_{2} & g_{3} & 0 & 0 & 0 & 0\\ 0 & 0 & g_{0} & g_{1} & g_{2} & g_{3} & 0 & 0\\ 0 & 0 & 0 & 0 & g_{0} & g_{1} & g_{2} & g_{3}\\ g_{2} & g_{3} & 0 & 0 & 0 & 0 & g_{0} & g_{1} \end{array}\right]\cdot \left(\begin{array}{c} f_{0}\\ f_{1}\\ f_{2}\\ f_{3}\\ f_{4}\\ f_{5}\\ f_{6}\\ f_{7} \end{array}\right)=\left(\begin{array}{c} y_{0}\\ y_{1}\\ y_{2}\\ y_{3}\\ y_{4}\\ y_{5}\\ y_{6}\\ y_{7} \end{array}\right)=\left(\begin{array}{c} c_{0}\\ c_{1}\\ c_{2}\\ c_{3}\\ d_{0}\\ d_{1}\\ d_{2}\\ d_{3} \end{array}\right)=Y$$

(13) B·F=Y

### 3.2. Multiscale Decomposition of the Wavelet Matrix

The wavelet matrix is used to explain the process of multiscale decomposition. Suppose that the original data to be analyzed consist of 128 points and that their corresponding DB4 wavelet matrix is also a 128th-order square matrix B[128,128]. *B* includes the scale matrix H[64,128] and wavelet matrix G[64,128], in which the *H* matrix is located in the upper half of the *B* matrix, and the *G* matrix is located in the lower half of the *B* matrix. The square brackets represent the number of rows and columns, as shown in

(14) $$B[128,128]=\left(\begin{array}{c} H[64,128]\\ G[64,128] \end{array}\right)$$

(15) $$F=B^{T}\cdot \left(\begin{array}{c} C_{1}\\ D_{1} \end{array}\right)=\left(\begin{array}{cc} H^{T} & G^{T} \end{array}\right)\cdot \left(\begin{array}{c} C_{1}\\ D_{1} \end{array}\right)=H^{T}C_{1}+G^{T}D_{1}=F_{1}+X_{1}$$

Inserting the relationships of $C_{1}$ and $D_{1}$ into this latter equation gives

(16) $$F=\left(H^{T}H+G^{T}G\right)\cdot F=H^{T}HF+G^{T}GF=F_{1}+X_{1}$$

In this formula, $H^{T}H$ is called the “low-frequency filter matrix” and $G^{T}G$ is called the “high-frequency filter matrix.” Taking the eighth-order DB4 orthogonal matrix as an example, because the B matrix is an orthogonal square matrix, we have

(17) $$B^{T}B=BB^{T}=E[128,128]$$

To calculate the next scale, we decompose the original data into a scale amplitude and a wavelet amplitude:

(18) $$B_{1}[64,64]=\left(\begin{array}{c} H_{1}[32,64]\\ G_{1}[32,64] \end{array}\right)$$

There are 64 points in total. From the wavelet amplitude, we can get the high-frequency wave $X_{1}$; because it is the highest frequency, there is no need for division. To decompose the lower frequency wave $F_{2}$, we start from the scale amplitude $C_{1}$, so we need to introduce the 64th-order DB4 wavelet matrix in Equation (18).

Note that the DB4 wavelet matrices of various scales are orthogonal matrices:

(19) $$\begin{array}{c} B^{T}\cdot B=E[128,128]\\ B_{1}{}^{T}\cdot B_{1}=E_{1}[64,64] \end{array}\}$$

where E is the unit matrix of order 128 and $E_{1}$ is the unit matrix of order 64. We regard the scale amplitude $C_{1}[64]$ as the original data, and we decompose and reconstruct it as follows:

(20) $$(B_{1}^{T}B_{1})\cdot C_{1}=(H_{1}^{T}H_{1}+G_{1}^{T}G_{1})\cdot C_{1}=F_{2}^{C}+X_{2}^{C}$$

In addition, note that $F_{2}^{C}[64]$ and $X_{2}^{C}[64]$ are the only data at a Scale 2 level, with only 64 points. After performing a further double expansion, the low-frequency wave $F_{2}[128]$ and high-frequency wave $X_{2}[128]$ under Scale 1 are obtained as

(21) $$H^{T}\cdot (F_{2}^{C}+X_{2}^{C})=F_{2}+X_{2}$$

Similarly, the wavelet decomposition of Scale 3 can be derived, and its function can be expressed as follows:

(22) $$F_{3}=H^{T}(H_{1}^{T}[H_{2}^{T}H_{2}]H_{1})H\cdot F={\left(H_{2}H_{1}H\right)}^{T}(H_{2}H_{1}H)\cdot F$$

(23) $$X_{3}=H^{T}(H_{1}^{T}[G_{2}^{T}G_{2}]H_{1})H\cdot F={\left(G_{2}H_{1}H\right)}^{T}(G_{2}H_{1}H)\cdot F$$

In this paper, the form of the DB4 wavelet matrix is expressed clearly by the method of the wavelet matrix. Luckily, in the analysis of discrete wavelets, DB6, DB8,……, DB20 and other DB 2N (N is a positive integer) can form an orthogonal B matrix according to the method, and realize the multiscale decomposition of the signals.

### 3.3. Extraction of the Nonstationary Wind Model

In the stationary wind speed model, when calculating the influence of wind load on the structure, it is usually assumed that the wind load consists of two parts: static action caused by mean wind and dynamic action caused by fluctuating wind. Respectively, these can expressed as

(24) $$U(t)=\bar{U}+u(t)$$

(25) $$V(t)=\bar{V}+v(t)$$

(26) $$W(t)=\bar{W}+w(t)$$

where $\bar{U},$ $\bar{V}$, and $\bar{W}$ represent the mean wind speed in the longitudinal, lateral, and vertical directions, respectively; and u(t), v(t), and w(t) are the corresponding zero-mean fluctuating wind.

In the nonstationary wind speed model, the wind model can be expressed as

(27) $$U(t)={\bar{U}}^{*}+u^{*}(t)$$

(28) $$V(t)={\bar{V}}^{*}+v^{*}(t)$$

(29) $$W(t)={\bar{W}}^{*}+w^{*}(t)$$

where ${\bar{U}}^{*}$*,* ${\bar{V}}^{*}$, and ${\bar{W}}^{*}$ represent the time-varying mean wind speed in the longitudinal, lateral, and vertical directions, respectively; and $u^{*}(t)$, $v^{*}(t)$, and $w^{*}(t)$ are the corresponding zero-mean fluctuating wind.

**Figure 5:**
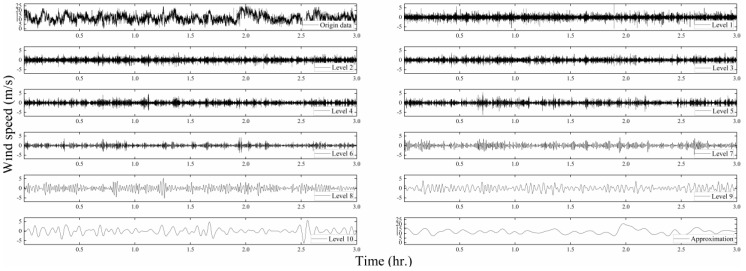
Wavelet-matrix transform (WMT) decomposition of the Lekima data.

**Figure 6:**
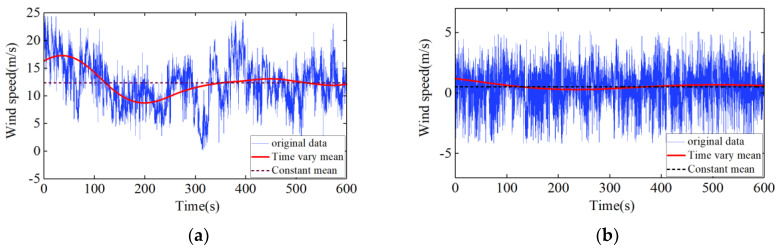
Comparison of the constant mean and time-varying mean wind speeds: (**a**) longitudinal component; (**b**) lateral component.

To extract the time-varying characteristics of the nonstationary wind speed, as shown in Figure 5, the abovementioned DB10 wavelet matrix multiscale method was used for a 10-level decomposition [6,21]. With the increase in level number, the filtered frequency component becomes smaller and the approximate value better represents the time-varying mean value. Figure 6 compares the Typhoon Lekima constant mean wind speed model and the time-varying mean wind speed model. It can be seen from Figure 6 that the time-varying mean wind speed calculated by using the wavelet matrix algorithm fluctuates around the constant mean wind speed calculated by the stationary wind speed model. Its 10-min constant mean along the wind speed is 12.33 m/s, the time-varying mean wind speed fluctuates between 8.7 and 17.2 m/s, and the crosswind speed is close to the constant mean speed. It can be seen that the time-varying mean wind speed can better reflect the trend of the nonstationary wind speed.

## 4. Field Measurements and Wind Characteristics

### 4.1. Turbulence Intensity

Turbulence intensity is an important characteristic to describe the variation in wind speed with time and space and the relative intensity of the fluctuating wind speed. Based on the turbulence intensity calculation formula of the stationary wind speed model, the time-varying mean wind speed of the nonstationary wind speed model and the corresponding fluctuating wind speed are substituted. The turbulence intensity of the nonstationary wind speed model, $I_{i}^{*}$, is expressed as the ratio of the standard deviation of the fluctuating wind speed to the interval T, the time-varying mean wind speed:

(30) $$I_{i}=\frac{\sigma_{i}}{\bar{U}},i=u,v$$

(31) $$I_{i}^{*}=\frac{\sigma_{i^{*}}}{{\bar{U}}^{*}},i=u,v$$

where $I_{i}$ and $I_{i}^{*}$ are the stationary wind speed model and nonstationary wind speed model turbulence intensities, respectively; $\sigma_{u}$ and $\sigma_{u^{*}}$ the corresponding standard deviations; *u* and *v* represents the bridge in the longitudinal and lateral, respectively; and, according to China’s code, *T* = 10 min this study. [14].

**Figure 7:**
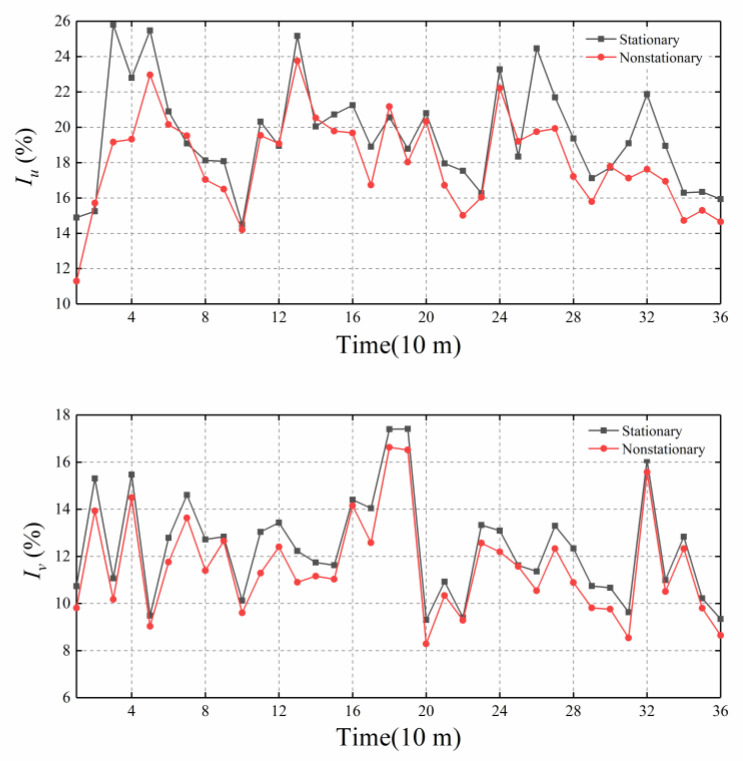
Comparison of the turbulence intensities of Typhoon Lekima with a 10-min duration.

It can be seen from Figure 7 that, in the typhoon period, the turbulence intensities $I_{u}^{*}$ and $I_{v}^{*}$ calculated based on the nonstationary wind speed model in the downwind and crosswind directions, respectively, are lower than those in the stationary wind speed models ($I_{u}$ and $I_{v}$), which are quite different in some periods, indicating that the results of the stationary wind speed model offer greater safety. The turbulence intensity along the wind direction is greater than that across the wind direction. Values of the average $\bar{I_{u}}$ and $\bar{I_{v}}$ of Typhoon Lekima based on the stationary wind speed model are 19.52% and 12.4%, respectively, while the values of average $\bar{I_{u}^{*}}$ and $\bar{I_{v}^{*}}$ based on the nonstationary wind speed model are 18.07% and 11.56%.

### 4.2. Gust Factor

In the traditional stationary model, the gust factor is defined as the ratio of the maximum gust wind speed during the gust duration $T_{g}$ to the mean wind speed in the basic time interval *T*. For the nonstationary gust factor, it is expressed as the maximum ratio of the mean value of the original wind speed to the mean value of the time-varying average wind speed during the gust duration. These are expressed as follows

(32) $$G_{u}(t_{g},T)=\frac{\max{\left[\bar{U}(t_{g})\right]}_{T}}{\bar{U}}$$

(33) $$G_{u}^{*}(t_{g},T)=\max{[\frac{\bar{U}(t_{g})}{{\tilde{U}}^{*}(t_{g})}]}_{T}$$

where $G_{u}(t_{g},T)$ and $G_{u}^{*}(t_{g},T)$ are the gust factors in the stationary model and the gust factors in the nonstationary model, respectively; $\bar{U}(t_{g})$ is the mean value of the original wind speed recorded during the gust duration $t_{g}$; and ${\tilde{U}}^{*}(t_{g})$ is the mean value of the variable mean wind speed during the gust duration $t_{g}$.

**Figure 8:**
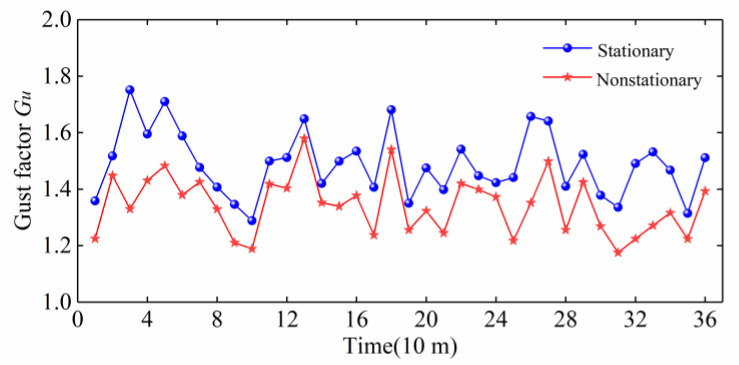
Comparison of the gust factors of Typhoon Lekima with a 10-min duration.

As shown in Figure 8, the gust factors of the stationary wind speed model and the nonstationary wind speed model are compared. It can be seen from that the gust factors of stationary wind speed model and nonstationary wind speed model have a similar fluctuation trend and amplitude size, but the gust factor of the stationary model is larger than that of the nonstationary model.

**Figure 9:**
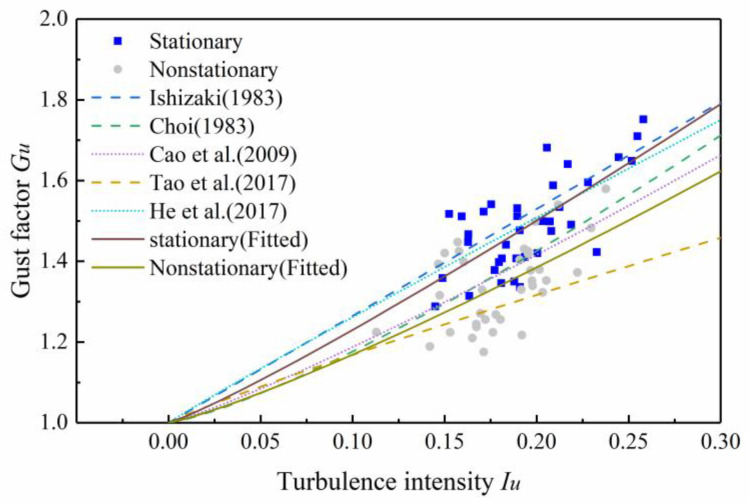
Stationary and nonstationary gust factors versus turbulence intensity.

Tao [21] and He [17] reported that the gust factor was closely related to the longitudinal turbulence intensity. Figure 9 shows the relationship between the gust factor and longitudinal turbulence intensity under the stationary model and nonstationary model.

Many scholars have fitted the relationship between gust factor and longitudinal turbulence intensity [26,27]. The expressions of the relationship between gust factor and the intensity of longitudinal turbulence are presented based on the linear model and the nonlinear model, which can be expressed in one equation as follows:

(34) $$G_{u}(t_{g},T)=1+k_{1}I_{u}^{k_{2}}In(\frac{T}{t_{g}})$$

where Ishizaki suggested $k_{1}$ = 0.5, $k_{2}$ = 1.0 for typhoons [28]; Choi suggested $k_{1}$ = 0.62, $k_{2}$ = 1.27 [29]; Cao suggested $k_{1}$ = 0.5, $k_{2}$ = 1.15 for Typhoon Maemi [30]; Tao et al. suggested $k_{1}$ = 0.26, $k_{2}$ = 0.91 for a nonstationary model [21]; and He et al. suggested $k_{1}$ = 0.45, $k_{2}$ = 0.96 for a nonstationary model [17].

**Figure 10:**
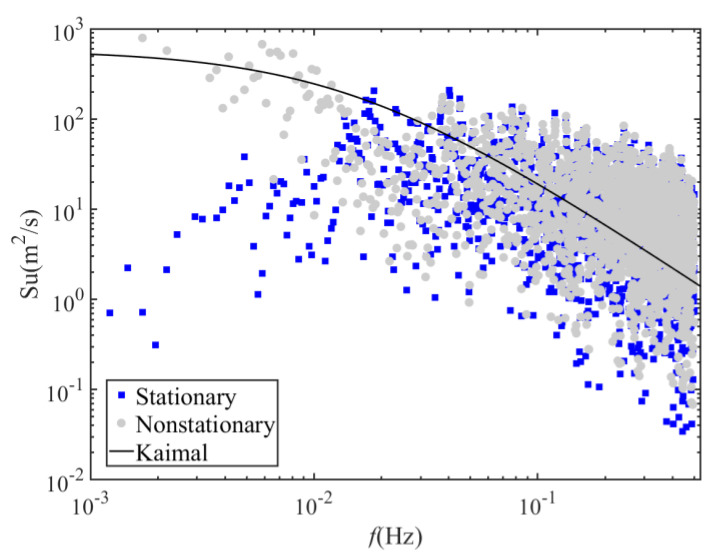
Comparison of the measured longitudinal spectra of Typhoon Lekima and the Kaimal spectrum.

The relationship between gust factor and turbulence intensity under typhoon Lekima were fitted for comparison. Where the stationary model suggests $k_{1}$ = 0.57, $k_{2}$ = 1.12 and the nonstationary model suggests $k_{1}$ = 0.49, $k_{2}$ = 1.18. As shown in Figure 10, the stationary model proposed in this study is similar to the He model, and the nonstationary model proposed in this study is similar to Cao model. The two fitting models proposed in this study can well describe the stationary and nonstationary relationships between the gust factor and turbulence intensity.

### 4.3. Turbulence Power Spectral Density

A fluctuating wind speed can be regarded as the superposition of vortices with different frequency components in space. To determine the contribution of different frequency components to the fluctuating wind energy, the power spectral density of the fluctuating wind can be obtained by using the Fourier transform of the autocorrelation function of the fluctuating wind. Through numerous field measurements of strong wind, many effective models of fluctuating wind spectra have been established. Among them, the Kaimal spectrum is adopted by China’s Code for “Wind-resistant Design of Highway Bridges” as the downwind fluctuating wind spectrum model [14]. For example, the expressions for the Kaimal spectrum corresponding to the stationary and nonstationary wind speed models are as follows:

(35) $$\frac{nS(n)}{u_{*}^{2}}=\frac{200(nz/\bar{U})}{{\left[1+50(nz/\bar{U})\right]}^{5/3}}$$

(36) $$\frac{nS^{*}(n)}{{\tilde{u}}_{*}^{2}}=\frac{200(nz/{\bar{U}}^{*})}{{\left[1+50(nz/{\bar{U}}^{*})\right]}^{5/3}}$$

In Equations (35) and (36), the power spectral density for stationary and nonstationary is expressed, respectively; *z* is the height from the ground; *n* is the natural frequency of fluctuating wind; and *u* and $\tilde{u}$ are the stationary and nonstationary friction wind speed, respectively. The friction wind speed cannot be measured directly but, because it is related to the mean square deviation of fluctuating wind, it can be obtained by the following energy normalization formulas:

(37) $$\sigma^{2}=6u_{*}^{2}$$

(38) $$\sigma_{*}^{2}=6{\bar{u}}_{*}^{2}$$

As shown in Figure 10, the low frequency part of the power spectral density values based on the stationary model are smaller than the power spectral density values based on the nonstationary model. The estimated power spectral density values based on the stationary and nonstationary models are slightly larger than the Kaimal spectrum of the high frequency part. In the low frequency part, the power spectral density values of the stationary model were smaller than the Kaimal spectrum, while power spectral density values of the nonstationary model were consistent with the Kaimal spectrum.

## 5. Buffeting Response Analysis of the Cables Based on the Measured Data

### 5.1. The RMS Value of the Measured Cable Acceleration Response

**Figure 11:**
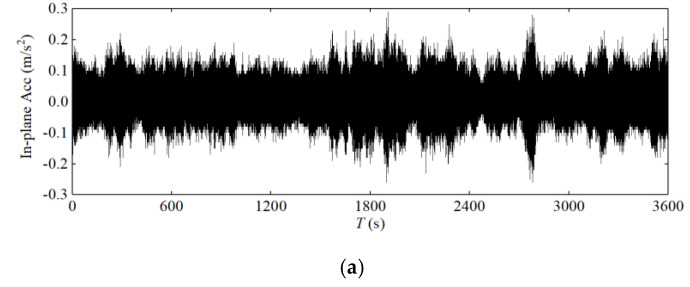

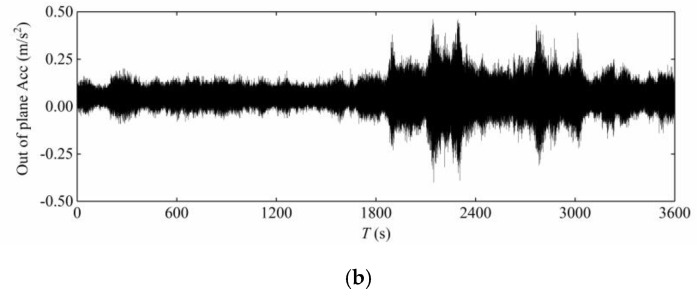
Measured acceleration of the upstream stay cable: (**a**) in plane; (**b**) out of plane.

**Figure 12:**
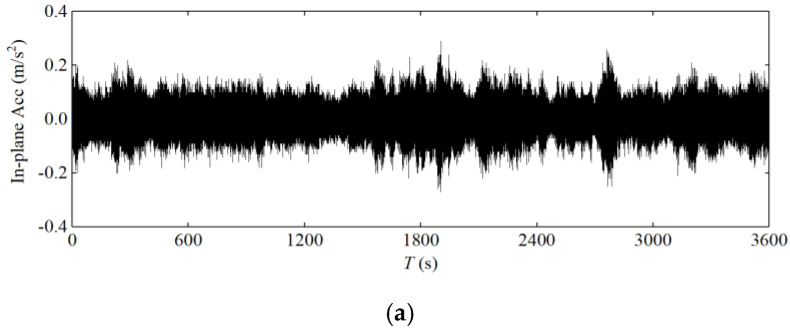

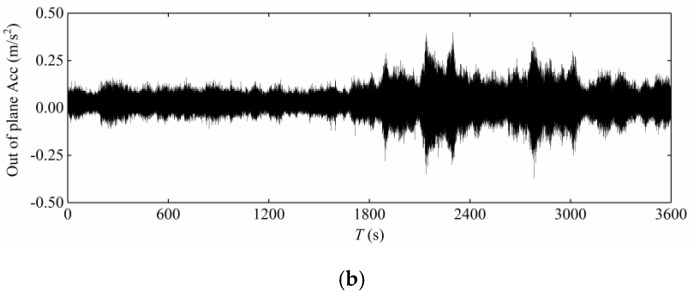
Measured acceleration of the downstream stay cable: (**a**) in plane; (**b**) out of plane.

As one of the main load-bearing components of cable-stayed bridges, the cables themselves are flexible components. With the rapid growth of the span of cable-stayed bridges, wind load plays a key role in cable design, as wind load can seriously endanger the durability and safety of cable-stayed bridges. Cables become a vulnerable component [31]. Therefore, field testing of cable vibration response is crucial [3]. In this study, the acceleration response of the longest stay cable of the Jintang Bridge was analysed. Figure 11 and Figure 12 shows the vibration response of this stay cable during Typhoon Lekima captured by the SHMS.

**Figure 13:**
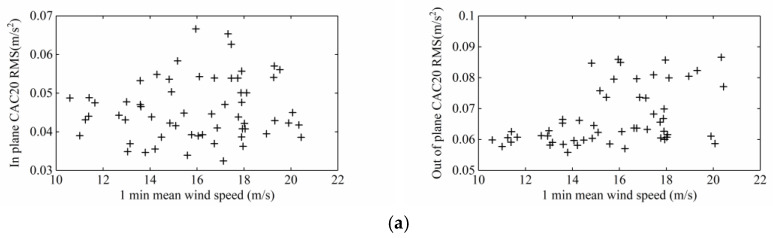

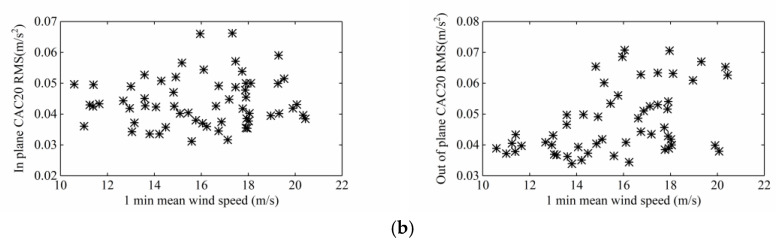
Root mean square stay cable acceleration response versus mean wind speed: (**a**) Upstream; (**b**) Downstream.

Figure 13 shows the relationship between the root mean square value of the 1-min-average acceleration and the 1-min-average wind speed. With the increase in wind speed, the in-plane and out-of-plane acceleration responses of the upstream and downstream stay cables exhibited a certain randomness, without obvious regularity, but the in-plane vibration was slightly greater than the out-of-plane vibration, and the vibration response was small and thus the control effect of the damper was very good.

### 5.2. Spectral Analysis of the Cable’s Measured Acceleration Response

**Figure 14:**
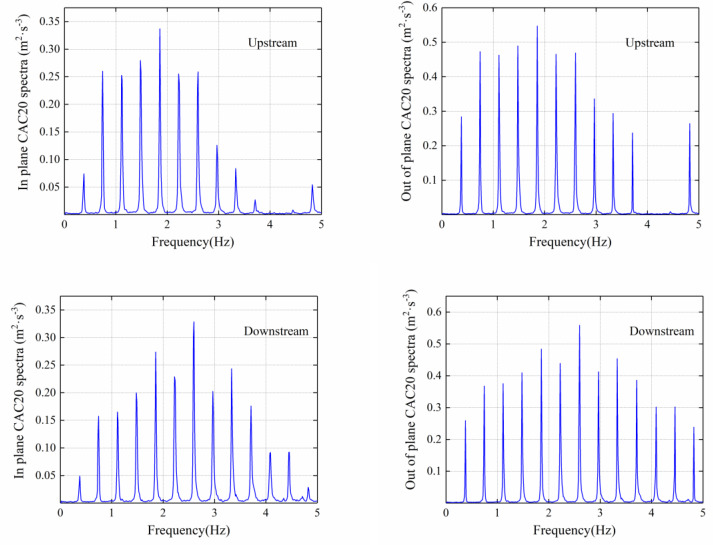
Spectra of the stay cable acceleration response.

The fast Fourier transform method was used to analyze the in-plane and out-of-plane acceleration response of the cable, and the results are shown in Figure 14. The amplitude of the in-plane and out-of-plane vibration on the same cable was different, but the vibration characteristics were very similar, with the frequency values of each order being nearly the same.

### 5.3. Wavelet Packet Energy Analysis of the Cable’s Measured Acceleration Response

To investigate the wavelet packet energy distribution of the stay cable of the Jintang Bridge during Typhoon Lekima, the acceleration response data collected by the longest stay cable sensor of the Jintang Bridge was used to calculate the wavelet packet energy spectrum over 1-min intervals. It has been pointed out that the multi-scale analysis of the bridge’s structure vibration response using DB wavelet can achieve good results [32,33,34]. The DB10 wavelet function was used, the decomposition level was 5, and the characteristic frequency band was the band of the first 32 largest energy coefficients.

**Figure 15:**
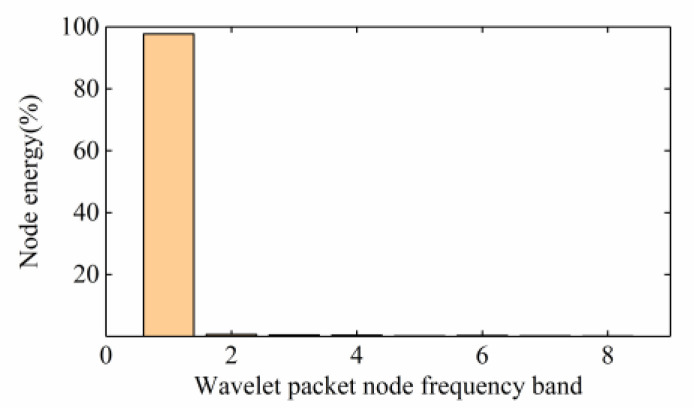
Energy distribution of the typhoon wavelet packet.

**Figure 16:**
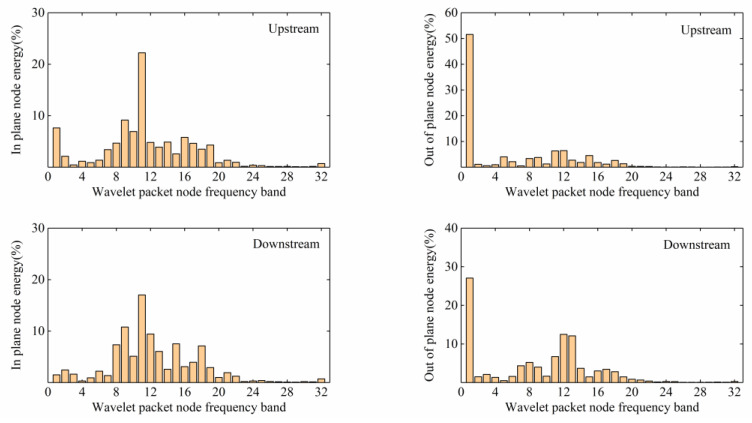
Energy distribution of the cable wavelet packet.

As shown in Figure 15, the energy of Typhoon Lekima was mainly distributed in the first frequency band (0–4 Hz), with an average energy ratio of 96.1%. The acceleration signals of the stay cables were analysed through wavelet energy analysis at the same time. As shown in Figure 16, the in plane vibration was mainly distributed in the 11th frequency band (31.25–34.375 Hz), with the upstream and downstream parts accounting for 51.6% and 27.1%, respectively, while the out of plane vibration was mainly distributed in the first frequency band, with the upstream and downstream parts accounting for 22.2% and 17.1%, respectively. It can be concluded that the out of plane vibration of the stay cables mainly occurred during the typhoon and these vibrations were of low frequency. In addition, the distribution of the typhoon energy in the high-frequency band was narrow, but the in-plane vibration energy of the stay cable was distributed over a wide range, which shows that a certain out of plane vibration mode of the stay cable existed in the 11th band.

**Figure 17:**
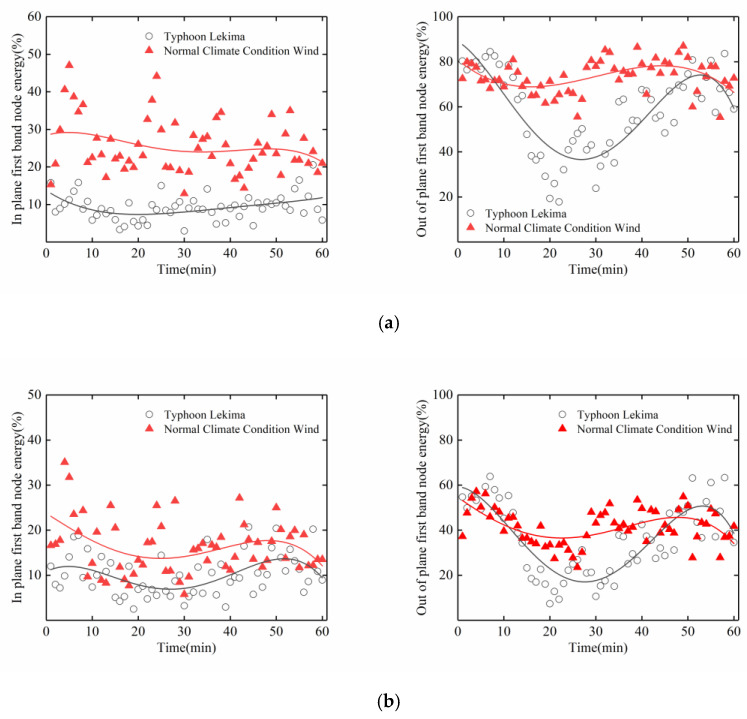
Energy distribution of the wavelet packet in the first frequency band of the cable: (**a**) Upstream; (**b**) Downstream.

**Figure 18:**
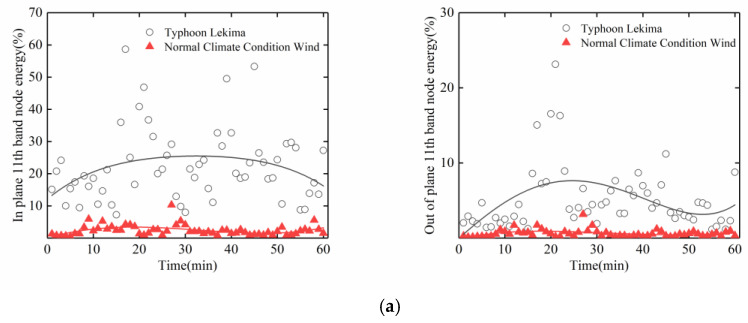

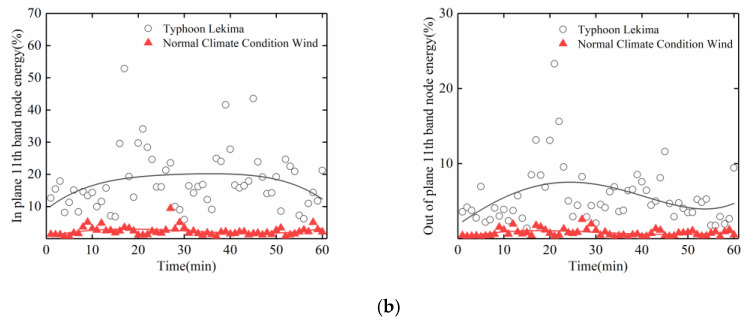
Energy distribution of wavelet packet in the 11th frequency band of the cable: (**a**) Upstream; (**b**) Downstream.

In view of the above energy analysis of the typhoon and stay cables, we again make a separate comparative analysis of the data in the first frequency band and the 11th frequency band between the period of Typhoon Lekima and the normal climate conditions wind period. It can be seen from Figure 17 and Figure 18 that, in the 1st frequency band, for the normal climate conditions, the wind period for the in-plane and out-of-plane vibrations of the stay cables upstream and downstream were higher than those during the typhoon period, whereas, in the 11th frequency band, for the typhoon period, the in-plane vibrations of the stay cables upstream and downstream were higher than those in the normal climate conditions wind period, which indicates that the high-order vibration of the stay cable is caused by the typhoon. Attention should therefore be paid to the high-order, vortex-induced vibrations of the stay cables, to avoid fatigue damage to the stay cables.

## 6. Conclusions

The nonstationary characteristics of Typhoon Lekima and the energy buffeting response distribution of the cable acceleration of the Jintang Bridge were analysed. The following conclusions can be drawn:

1. The turbulence intensity in the nonstationary model is smaller than that in the stationary model, and the safety factor of the stationary model is higher, which is relatively conservative. In the engineering application, the stationary model can be used for design reference.
2. The two fitting models proposed in this study can well describe the stationary and nonstationary relationships between the gust factor and turbulence intensity. The stationary model proposed in this study is similar to the He model, and the nonstationary model proposed in this study is similar to the Cao model.
3. In the low frequency part, the power spectral density values of the stationary model were smaller than the Kaimal spectrum, while the power spectral density values of the nonstationary model were consistent with the Kaimal spectrum. It shows that the nonstationary model is more suitable for wind spectrum estimation.
4. The measured vibration characteristics of the upstream and downstream cables have obvious regularity. The in-plane vibration or out of plane vibration of the same cable is very similar, and the out of plane vibration is greater than the in-plane vibration, which shows that the damper has a good effect on suppressing the in-plane vibration.
5. The energy distribution in and out of the cable plane are different between the typhoon period and the normal climate condition wind period. It is mainly concentrated in the first frequency band (0–3.125 Hz) and the 11th frequency band (31.25–34.375 Hz); so, we should pay special attention to the high-order, vortex-induced vibration of the cables during a typhoon.

In general, considering the difference in wind environments in different regions, it is necessary to study the extreme value distribution of the nonstationary buffeting response of long-span, cable-stayed bridges in a sea environment, and form the engineering practice that can accurately simulate the buffeting response under extreme wind conditions to guide the design.

## Figures and Tables

**Table 1:** Wireless acceleration node TZT3805.

Project	Technical Standard
Number of channels	Single and double
Sampling frequency	200 Hz, adjustable
Frequency response	78 Hz DC
Range	2 g
Communication mode	4G
Sampling method	Timing, interval, trigger, continuous acquisition
Data storage	Cloud server
Work time	Solar energy or 220 V AC, long-term monitoring
Work temperature	−10 °C to +80 °C
Relative humidity	20%–85%
IP protection level	IR67
IR protection level	IK10
